# Strengthening methods for tracking adaptations and modifications to implementation strategies

**DOI:** 10.1186/s12874-021-01326-6

**Published:** 2021-06-26

**Authors:** Amber D. Haley, Byron J. Powell, Callie Walsh-Bailey, Molly Krancari, Inga Gruß, Christopher M. Shea, Arwen Bunce, Miguel Marino, Leah Frerichs, Kristen Hassmiller Lich, Rachel Gold

**Affiliations:** 1 Gillings School of Global Public Health, University of North Carolina at Chapel Hill, 1105C McGavran-Greenberg Hall, Chapel Hill, NC 27599-7411 USA; 2 George Warren Brown School, Washington University in St. Louis, 1 Brookings Dr, St. Louis, MO 63130 USA; 3 OCHIN, Inc, 1881 SW Naito Pkwy, Portland, OR 97201 USA; 4 Kaiser Permanente, Center for Health Research, 3800 N. Interstate Ave, Portland, OR 97227 USA; 5 Oregon Health and Science University, 3181 S.W. Sam Jackson Park Rd., Portland, OR 97239 USA

**Keywords:** Implementation strategies, Implementation context, Modification and adaptation, Reporting

## Abstract

**Background:**

Developing effective implementation strategies requires adequate tracking and reporting on their application. Guidelines exist for defining and reporting on implementation strategy characteristics, but not for describing how strategies are adapted and modified in practice. We built on existing implementation science methods to provide novel methods for tracking strategy modifications.

**Methods:**

These methods were developed within a stepped-wedge trial of an implementation strategy package designed to help community clinics adopt social determinants of health-related activities: in brief, an ‘Implementation Support Team’ supports clinics through a multi-step process. These methods involve five components: 1) describe planned strategy; 2) track its use; 3) monitor barriers; 4) describe modifications; and 5) identify / describe new strategies. We used the Expert Recommendations for Implementing Change taxonomy to categorize strategies, Proctor et al.’s reporting framework to describe them, the Consolidated Framework for Implementation Research to code barriers / contextual factors necessitating modifications, and elements of the Framework for Reporting Adaptations and Modifications-Enhanced to describe strategy modifications.

**Results:**

We present three examples of the use of these methods: 1) modifications made to a facilitation-focused strategy (clinics reported that certain meetings were too frequent, so their frequency was reduced in subsequent wedges); 2) a clinic-level strategy addition which involved connecting one study clinic seeking help with community health worker-related workflows to another that already had such a workflow in place; 3) a study-level strategy addition which involved providing assistance in overcoming previously encountered (rather than de novo) challenges.

**Conclusions:**

These methods for tracking modifications made to implementation strategies build on existing methods, frameworks, and guidelines; however, as none of these were a perfect fit, we made additions to several frameworks as indicated, and used certain frameworks’ components selectively. While these methods are time-intensive, and more work is needed to streamline them, they are among the first such methods presented to implementation science. As such, they may be used in research on assessing effective strategy modifications and for replication and scale-up of effective strategies. We present these methods to guide others seeking to document implementation strategies and modifications to their studies.

**Trial registration:**

clinicaltrials.gov ID: NCT03607617 (first posted 31/07/2018).

## Contribution to the literature

- Tracking adaptations and modifications made to implementation strategies and key factors driving the decisions to modify can be crucial for assessing the impact of implementation strategies and replicating effective strategies.
- Despite advances in detailed tracking methods in implementation studies, little guidance exists for tracking adaptations and modifications made to implementation strategies.
- These methods outline a process for tracking adaptations and modifications made to implementation strategies, which build on existing tracking methods, implementation frameworks, and reporting guidelines.

## Background

*Implementation strategies* are actions or processes used to increase interventions’ uptake and sustainment [1]. Developing generalizable knowledge about these strategies requires carefully tracking and reporting on how they are applied. Several related guidelines exist; for example, Powell et al. (2015) provide a list of standardized implementation strategy labels and definitions, and Proctor et al. (2013) provide guidelines for reporting implementation strategies in sufficient detail to ensure they can be replicated in research and practice [2,3]. In addition, a handful of studies have developed and tested methods for tracking and reporting implementation strategies, [2, 4–9] including: tracking logs that are completed by clinicians conducting implementation activities [9]; a system for research teams to track and code implementation strategies in alignment with Proctor et al.’s reporting recommendations [2, 7]; and logs completed by stakeholders involved in the implementation process to report on implementation strategies [10].

Despite these efforts, implementation research and practice literature often lacks sufficient detail on how implementation strategies were operationalized, how and why they worked (or failed), and how to replicate or refine such strategies in future uses [11–13]. Notably, studies of implementation strategies’ effectiveness often fail to document adaptations and modifications made to these strategies. While methods exist for tracking and reporting implementation strategies, as described above, there is a dearth of methods for identifying and describing modifications made to such strategies. Yet given the dynamic nature of implementation, strategy modifications may be necessary based on implementation context [14–19]. The Framework for Reporting Adaptations and Modifications to Evidence-Based Interventions (FRAME) provides guidance on how to track adaptations and modifications made to clinical interventions [20], but additional work is needed to examine how this framework might be applied to implementation strategies. Based on the definitions in FRAME, we use *adaptation* to refer to “thoughtful or deliberate alterations” made to implementation strategies “with the goal of improving its fit or effectiveness in a given context [20].” *Modification* encompasses a broad range of changes to strategies including adaptations, additions, and unanticipated, iterative changes that emerged naturally throughout the implementation process [20]. Finley et al. (2018) present one potential method, as structured reflection sessions throughout implementation show promise in documenting both modifications and associated contextual factors [21]. There is a clear need to further identify and test methods for documenting implementation strategy adaptations and modifications, as such methods are necessary to determine how and why implementation strategies deviate from plans and when such deviations are necessary. This knowledge is essential for replicating implementation studies’ results and disseminating best practices across settings.

This paper builds on existing methods for tracking implementation strategies to provide novel methods for tracking strategy adaptations and modifications [7, 9, 10, 21]. These methods include prospective tracking and coding of originally planned implementation strategies (i.e., those in the study protocol), and how those strategies were adapted and modified throughout a study. As little guidance for tracking modifications made to implementation strategies has been previously published, this paper is intended to help others hoping to track such modifications.

## Methods

### Study context

The methods presented here were developed in the context of a mixed methods, pragmatic, stepped-wedge, cluster-randomized trial, with a hybrid type 3 implementation-effectiveness design. The parent trial (funded in the U.S. by NIDDK 5R18DK114701) is assessing the effectiveness of an implementation strategy package designed to help community health centers (CHCs) adopt social determinants of health (SDH) screening and referral activities, called ‘SDH activities’ [22]. In each of six sequential wedges (referred to throughout this paper as ‘wedge 1,’ ‘wedge 2,’ etc.), up to five CHC clinics receive 6 months of technical assistance from a multi-disciplinary ‘Implementation Support Team’ with an electronic health record (EHR) trainer, practice coach, and SDH expert. The Implementation Support Team guides the clinics through a multi-step process called the ‘Clinic Action Plan,’ developed based on lessons learned from a pilot study [23] and refined from wedge to wedge. Implementation strategies are provided to support each step, as described in Gold et al. (2019) and summarized Table 4 in Appendix [22]. Per study protocol, any aspects of the planned implementation support could be modified to meet individual clinics’ needs, where feasible [22]. Modifications could be made in response to an individual clinic’s context *(clinic-level),* or perceived need to change the strategies delivered to all clinics *(study-level).*

### Tracking process

We developed the methods presented here to fully capture and describe the implementation support provided to each study clinic, by systematically tracking the implementation strategies used, and modifications made to the originally planned strategies. To develop these methods, we identified processes and data sources presumed critical to tracking implementation strategies and their adaptations using existing methods for tracking implementation strategies [7, 9, 10], guidance from implementation frameworks such as the Consolidated Framework for Implementation Research (CFIR) [24], and reporting guidelines including the Framework for Reporting Adaptations and Modifications-Enhanced (FRAME) [20] and Proctor et al.’s (2013) reporting framework [2].

**Fig. 1:**
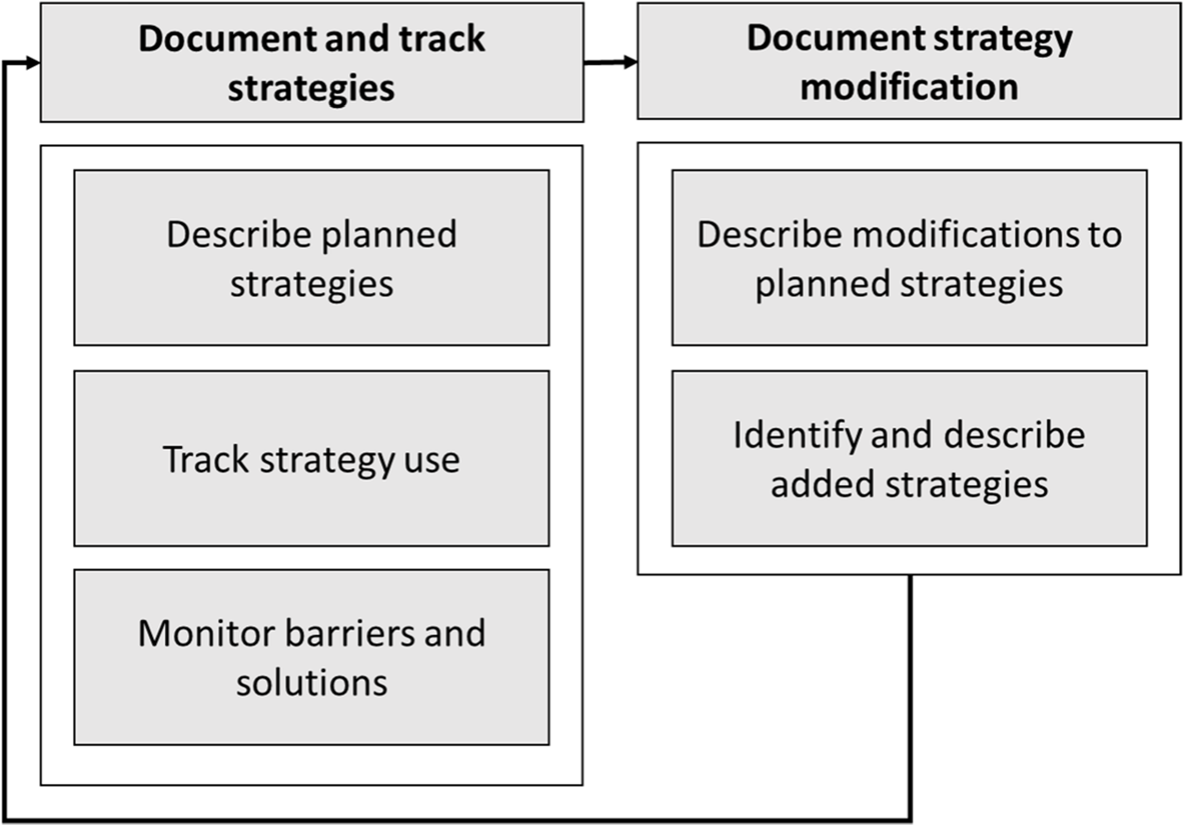
Five components tracked by these methods

These methods involve five components that are presented sequentially here, but in practice were often iterative or overlapping: 1) *describe each planned strategy* in detail; 2) *track* how the strategies are used; 3) *monitor barriers* and contextual factors that could impact strategy modification; 4) *describe modifications* made to planned strategies in response to barriers and contextual factors; and 5) *identify and describe new strategies* added during the study period. These are shown in Fig. 1 and described in detail below. To collect the data needed for these components, we drew on and augmented the rigorous documentation already planned as part of the parent trial. This tracking effort includes strategies provided to the parent study clinics by the research team; it is not intended to capture strategies initiated by the clinics themselves in the course of study participation.

### Describe planned strategies

We described all implementation strategies included in the study in detail to monitor deviations from their intended application. We used the Expert Recommendations for Implementing Change (ERIC) taxonomy of 73 discrete implementation strategies, and research building on ERIC, to *categorize* these strategies [3, 8]. We then *described* each strategy using Proctor et al.’s (2013) reporting framework, which recommends documenting a given strategy’s *actor*, *action*, *dose*, *temporality*, *action target*, and *justification* [2]. We drew on the parent study’s protocol and study materials to describe each strategy [22], named each strategy using ERIC, defined it based on study materials, and described each facet using Proctor’s framework. Members of the study team then verified the detailed list of planned implementation strategies.

### Track strategy use

We tracked the use of implementation strategies with each CHC clinic, with details on when and how the strategies were used, to identify modifications made to the strategies and / or differences between what was originally planned and what was delivered. To do so, the Implementation Support Team closely tracked and documented each study clinic’s implementation progress on a weekly basis. The research team monitored these notes weekly for changes and synthesized the documentation quarterly using the fields shown in Table 1. These data included documentation of regularly scheduled meetings with study clinics, dates when clinics reached critical milestones, materials sent to or received from the clinics, and clinic goals. The Implementation Support Team also included support that was provided to the study clinics beyond what was planned in the original intervention.

**Table 1:** Data elements tracked in original plan

Documentation tool	Data elements
Clinic Action Plan (CAP) Tracker: Which action plan step and task had been completed by each clinic; noted if targeted screening population had changed When collected: Form completed by clinics prior to each study clinic check-in call (2x/month)	Date of check-in
	Date each CAP step completed
	Completion status of each CAP step
	Any implementation challenges*
	Changes in population targeted for SDH screening
	Notes**
Check-In Tracker: Overview of the content planned and outcomes from each of the check-in meetings with individual clinics When collected: Completed after each clinic check-in.	Date of check-in
	Planned agenda
	Led by which team members
	Attended by which clinic staff
	Attended by which IST staff
	Summary of discussion
	Next steps / action items
	Comments / clarifications
Peer Support Call Tracker: The planned content and outcomes from the peer support meetings with all clinics in a given wedge When collected: Planned content – prior to each call, at the IST meeting; outcomes – at the IST meeting after the Office Hours	Date
	Planned agenda
	Led by which team members
	Attended by which clinic staff
	Summary of discussion
	Next steps/ action items*
	Comments
Materials Tracker: Materials sent to clinics and received from clinics When collected: Updated on an ongoing basis after materials were sent out to each clinic	Materials sent to clinics
	Dates materials were sent
	Materials received from each clinic*
	Date materials were received*
Other Support Tracker: Any other support provided to clinic outside of what we had already planned All “additional support” beyond what we had originally planned to provide to clinics, such as requests from clinics or additional support that our interactions with the clinics suggested would be helpful for individual clinics. When collected: At the IST meeting or during each clinic check-in	Topic Planned / Content
	Led by which team members
	Attended by which clinic staff
	Summary of discussion
	Comments
	Consultations outside of IST
	Next steps / action items
	Date of support received
	Led by which team members
	Clinic participants involved
	Comments
Clinic Update Tracker:* Clinic overview used for discussion during the weekly IST meetings Note: This replaced the Implementation Timeline and Monthly Reports When collected: Completed weekly for each clinic by the practice coach and EHR trainer prior to IST meetings and updated during weekly IST meetings	Date of Check-In
	Overview of clinic
	Clinic step/progress
	On track
	Successes
	Challenges
	Clinic goals
	Action items
	Questions from IST
	Next Agenda/Date
	Notes for Next Meeting
Implementation Timeline:** Iterative bird’s-eye planning tool to support each of the clinics throughout the intervention When collected: Sometimes pre-populated by IST members in advance of IST meeting but usually updated at each IST meeting (1x/week)	Step
	Date
	Type of clinic meeting
	Topic
	Content/Planned Agenda
	Led by which team members
Monthly Reports:** Overview of the clinic’s progress through Steps 1–5, and what they requested of the IST When collected: Summary was created after monthly activities were complete and presented to the IST and sent to all clinics the first week of the following month	Date
	Clinic name
	Progress made on each step
	Requested information from each clinic

*Abbreviations:* * addition made during the study, ** removed during the study

### Track barriers and solutions

We monitored discussions of clinics’ contextual factors or barriers, and of decisions made about adapting and modifying implementation strategies in response to these factors. We drew from detailed notes and transcripts from meetings with the Implementation Support Team and each clinic, and notes and recordings of weekly Implementation Support Team meetings. These sources enabled identifying the rationale for modifying implementation strategies, and whether it occurred at the clinic or study level. We then used the Consolidated Framework for Implementation Research (CFIR) to code these barriers / contextual factors [24]. The CFIR provides a comprehensive list of contextual factors that may impact implementation success, categorized as associated with: Outer Setting, Inner Setting, Characteristics of Individuals, and Characteristics of the Intervention, all with extensive sub-categories (see Additional File 3 of Damschroder et al., 2009). We did not use the Process constructs from CFIR due to their potential overlap with the ERIC taxonomy [3, 24].

### Describe modifications to planned strategies

We described adaptations and modifications made to strategies by documenting any deviations from the planned process*.* To document these, we drew on the detailed descriptions of planned strategies, strategy use, and barriers and solutions as described above. We then ensured that our approach to documenting these modifications was consistent with existing methods by building on published tracking methods [7, 9] and coding taxonomies [2, 3, 20, 24], by including elements of these taxonomies that we considered relevant to documenting implementation strategy modifications, as follows (Table 2).

**Table 2:** Data elements tracked to capture modifications to implementation strategies

Data element	Description
**Frame**	
Modification	Briefly describe the modification
When did modification occur	Note when the modification was made (e.g week, month or wedge, cohort)
Who made decision to modify	Note implementation team, practice coach, clinic, or specify other
Goal of modification	Describe anticipated change as a result of the modification
Nature of modification	Select tweaking/refining, shortening, lengthening, reordering, removing/skipping
Reason for modification	Summary of challenges the modification was meant to address, use CFIR categories, and FRAME categories as additions
Source information for reason	Note specific source of information for the reason
**CFIR / ERIC**	
CFIR domain	Reason, barrier or determinant coded using CFIR
ERIC category	Strategy coded to broad category using ERIC
ERIC implementation strategy	Strategy coded to specific implementation strategy
**Proctor**	
Primary actor	Who enacts the strategy? Indicate the position of actor if possible
Supporting actor(s)	Any internal or external person who is helping the primary actor
Action	Provide a detailed description of the action taken by each actor.
Format	Learning session, coaching call, email or other informal contact
Dose	Frequency, duration, time required, scaled over time; start and end dates
Temporality	Does this strategy need to occur in sequence with other strategies
Justification	State reason strategy being used
Action Target	Person or groups whose knowledge, attitudes, or behavior should change, and state change
Outcome	List any outcome reported that would show that the strategy had an effect
Enacted	Was the strategy used
**Notes**	

We used elements of the Framework for Reporting Adaptations and Modifications-Enhanced (FRAME) [20] to describe strategy modifications, an expansion of Stirman et al.’s review [25]. FRAME describes elements that should be considered when tracking modifications and adaptations made to interventions as they are implemented. Here, we explored FRAME as a tool for reporting modifications to implementation strategies, rather than the intervention. We used many of FRAME’s reporting elements, and added elements from implementation frameworks or project-specific language as needed.

We included FRAME elements to describe the *nature* of the modification (e.g. adding, tweaking or refining, lengthening or shortening, reordering strategies, or removing or skipping elements), *when* the modification occurred (e.g. pre-implementation, or stage in the study), *who* participated in the decision to modify (e.g. Implementation Support Team, practice coach, clinic champion), and the *reason* why the modification was made (e.g. staffing, available resources, competing demands). FRAME also includes *level of delivery*; here, this meant whether the modification was at the clinic or study level. For strategies that were not enacted (e.g., because a given clinic did not get to the implementation support within the study period) we coded the *nature* of the modification as *removing or skipping* elements.

We used CFIR to augment the documentation of the *reason* for a given strategy modification. In this study, the *reasons* for modifications were often implementation barriers. While the FRAME categories were a useful starting point, CFIR is a more comprehensive framework to describe implementation barriers. Using CFIR for *reasons* also allowed for greater consistency of coding, as it was also used to identify barriers and contextual factors earlier in the process. Strategies were often added to address common implementation barriers. For example, if a study clinic had not planned for SDH screening, it was coded as *planning*; if clinic staff had inadequate knowledge about SDH screening, it was coded as *access to knowledge & information*; and if limited resources were dedicated to implementing SDH screening, it was coded as *available resources*.

Several elements of FRAME were considered not applicable, or unlikely to vary across modifications. For example, all modifications were considered *content* modifications (rather than *contextual* or *evaluation* modifications). We did not code for the *relationship to fidelity* or whether modifications should be considered *cultural*. It was not appropriate to consider fidelity to planned strategies as the study design intentionally allowed for modification. Guidelines for fidelity-consistent modifications were not developed for the strategies included in this intervention because the implementation support was designed to be adaptive and core elements were not yet known. Tracking modifications in response to culture was not appropriate given the focus on modifications to strategies rather than the intervention and the limited cultural variation in the study context and population. This element was added to FRAME to capture modifications made to interventions that are implemented in cultures different from where the intervention was developed. This was not applicable to our study.

### Identify and describe added strategies

The prior four components were used to track strategies that were planned and revised. However, unplanned strategies may also be added throughout an implementation process, which require slightly different tracking methods. For added strategies, we begin by populating elements of FRAME to *describe the addition*. Once these strategies are added, they can also be tracked to understand if they are used as intended. We track added strategies for subsequent modification by completing each component of the process to *describe the added strategy*, *track strategy use*, *monitor barriers and solutions*, and *describe any modifications* to the strategies as planned.

We identified strategies added for a given clinic using a separate tracking tool (Table 1), and strategies added at the study level using notes from Implementation Support Team meetings and intervention materials. We then briefly described the added strategy based on FRAME (Table 2), and coded it using the ERIC taxonomy and the Proctor reporting guidelines. Study-level additions were then included in the tracking of planned strategies and monitored as such in subsequent use.

## Results

This five-component process for tracking modifications made to implementation strategies in the context of an implementation study leveraged existing implementation frameworks, reporting guidelines, and methods for tracking implementation strategies. Clinic-level modifications were often based on clinic context and implementation needs; study-level modifications were often based on lessons learned over the course of the study, and were applied to clinics in subsequent wedges. Table 3 gives examples of the use of these methods.

**Table 3:** Examples of implementation strategy modifications

Data element	Example 1: study-level (shortening)	Example 2: clinic-level (addition)	Example 3: study-level (addition)
**Frame**			
Modification	Reduced frequency of peer support meetings from 1x/month to 1x/2 months	Additional information shared between clinics within a wedge	Additional questions in the assessment organizations complete at baseline
When did modification occur	After wedge 1	Within wedge 3	After wedge 2
Who made decision to modify	IST	Practice coach	IST
Goal of modification	Increase acceptability of the implementation effort	Improve outcomes	Improve fit
Nature of modification	Shortening, reduced frequency	Adding elements	Adding elements
Reason for modification	Organizational	Organizational, Available Resources, Staffing	Organizational, Context
Source information for reason	Based on discussion during IST meetings	Stated by clinic during check in meeting	Based on discussion during IST meetings
**CFIR / ERIC**			
CFIR domain	Access to Knowledge and Information	Available Resources	Readiness for implementation
ERIC category	Provide Interactive Assistance	Develop Stakeholder Interrelationships	Use Evaluative and Iterative Strategies
ERIC implementation strategy	Facilitation	Capture and share local knowledge	Assess for readiness and identify barriers and facilitators
**Proctor**			
Primary actor	Practice coach	Practice coach requests information from clinic with expertise	Practice coach
Supporting actor(s)	Project champions	Project champion at study clinic, staff at peer clinic	IST, project champion
Action	Meetings which include project champions and members of the implementation teams of all clinics within a study wedge. Practice coach facilitates meetings. Project champions attend meetings and share information between clinics	Practice coach requests information from clinic with expertise. Clinic with expertise shares knowledge, clinic seeking expertise reviews and uses the information	IST members modified the baseline assessment to include questions about determine whether aspects of the intervention have already been implemented at the clinic. Project champion completes the assessment. Practice coach uses the assessment to plan implementation support
Format	Virtual meeting	Email to request information, word document to share information, follow-up meeting to discuss information	Learning session, coaching call, email or other informal contact
Dose	Once a month	1 time	1 time
Temporality	Throughout the study	Prior to step 3	Prior to step 1
Justification	Pragmatic justification – meeting frequency should be feasible and acceptable to study participants	Pragmatic justification - peer-to-peer learning can be effective where there is no empirical evidence is limited	Pragmatic justification - understanding context can inform facilitation efforts
Action target	Clinic champion knowledge and self-efficacy	Clinic champion has increased knowledge of the role of community health worker	Practice coach has increased knowledge about clinic context prior providing implementation support
Outcome	Improved implementation through increased knowledge	Clinic champion is prepared to work within the clinic to develop an appropriate staffing plan	Practice coach feels more prepared to provide implementation support appropriate for clinic context
Enacted	Yes	Yes	Yes
**Notes**			

Example one is based on a facilitation strategy. Members of the Implementation Support Team conducted virtual meetings with project champions from all clinics in a given wedge. When working with the first set of study clinics, these meetings took place once a month throughout the support period, and were designed to improve implementation by increasing champion knowledge and self-efficacy and improving readiness. By *tracking strategy use*, we identified a change to this strategy between wedge 1 and wedge 2 of the parent study. To understand the reason for this change, we used process data from clinic interactions and internal meetings of the study team to *track barriers and solutions*. Several of these clinics reported that the meetings were too frequent, so the study team decided to reduce the frequency of these meetings in subsequent wedges. We used the description of the strategy, the tracking of the strategy use, and the tracking of barriers and solutions to *describe modifications* to the implementation strategy using elements of FRAME.

Examples two and three illustrate additions made to planned strategies. We used the *tracking of strategy use*, and the *tracking of barriers and solutions* to *identify the added strategy*. As part of tracking strategy use, the Implementation Support Team listed any “other support” provided to the clinics. This includes strategies that were not part of the planned study activities for that wedge. Data from this tracker showed that the practice coach connected a clinic in wedge three and a clinic from a prior wedge to share information. Additional process data to track barriers and solutions showed that the clinic wanted to identify and train appropriate staff to conduct screening and develop new workflows as part of developing the implementation plan. This clinic expressed a need to better understand the potential role of community health workers in this process. The clinic champion requested additional information about the job description of the community health workers at a peer clinic enrolled in the study. The practice coach contacted the clinic and requested that they share the job description for community health workers. We describe this strategy modification using FRAME, and *describe the strategy* in detail using the ERIC taxonomy and Proctor et al. reporting framework.

Example three illustrates a modification made by adding a strategy between wedges. Again, we used information from an earlier component in the process to identify and understand this added strategy. Process data from Implementation Support Team meetings showed that several study clinics were not taking on SDH activities de novo; many had attempted to do so in the past, and sought assistance in overcoming previously encountered challenges. To address this, the Implementation Support Team added questions to the study’s baseline survey to assess clinics’ past experience with SDH implementation, and factors that might impact the clinic’s ability to initiate, expand, or improve such activities. This strategy addition was administered pre-wedge, to improve the fit of future implementation strategies (Table 3).

## Discussion

This approach contributes to growing body of research to address calls for improved reporting of implementation interventions and strategies [2, 11, 19, 26]. Systematic reviews of implementation studies show that strategies are often not reported in sufficient detail to describe what was planned as part of the study design and whether strategies were executed as intended [27, 28]. This imprecise reporting hinders our ability to evaluate the impact of implementation strategies within and across studies, and make incremental improvements or refinements to strategies to improve their impact.

These methods outline a process for tracking adaptations and modifications made to implementation strategies, which build on existing tracking methods, implementation frameworks, and reporting guidelines. Integrating existing frameworks based on study context allowed for the potential to compare across studies, and to build on previous work to further refine the application of these frameworks for future research. No framework is comprehensive for all contexts, however, and each contains elements that are not applicable in particular contexts. Several challenges arose in applying and integrating these frameworks, as described below.

Although the selected frameworks generally suited the purposes of the study, we made additions to several frameworks. In the parent study, developing and adapting workflows was a key implementation strategy. This strategy is not part of the ERIC taxonomy. For this reason, we used suggested additions to the ERIC compilation as identified by Perry and colleagues [8]. We also added two components to the Proctor framework: ‘Supporting Actor’ (any other person who might need to be involved to ensure the strategy was completed other than the ‘Actor’), and Format of Strategy Delivery, to clarify mode of delivery of strategies. Supporting Actor provided additional detail where the primary actor of a strategy was external to the organization and the purpose of the strategy was to create change within the organization. It was useful to define the roles of both internal or external actors. We added ‘Format of Strategy Delivery’ to document changes from the planned mode of delivery: for example, steps to develop a clinic’s implementation plans were often completed during meetings, rather than in written format, as planned. This could be a critical detail to ensure a strategy’s replication, particularly where facilitation is a key implementation strategy.

In the application of these methods, there was overlap in elements of several frameworks. We did not use the CFIR ‘Process’ domain, as it was redundant with the ERIC documentation of implementation strategies. CFIR components were applied to describe both implementation barriers and reasons for strategy modifications. We found that the reasons for strategy modification were best described using CFIR’s comprehensive overview of the multi-level implementation determinants. We augmented CFIR categories with FRAME as needed. For example, we found that CFIR provided limited detail for describing barriers related to workforce; we could only code barriers related to insufficient workforce or staff turnover using the CFIR category *available resources*. Here, FRAME offered additional detail, with a sub-category *staffing*.

FRAME also provided elements for documenting modifications made to implementation strategies for individual clinics and at the study level. This was useful given the dynamic nature of the study design. While we used FRAME’s general categories on *when* the modification occurred, *who* participated in the decision to modify, the *nature* of the modification, and the *reason* why the modification was made, we generally used either a subset of the codes within these categories, or developed new codes. Additional research is needed to explore the application of FRAME to implementation strategies.

We selected these frameworks primarily based on their usability and applicability to the parent study [29, 30]. Future users of the methods presented here should consider whether other frameworks and data sources are a better fit in other contexts. For example, CFIR represents one of many determinant frameworks [31]. Alternatives include the Theoretical Domains Framework [32, 33] or the Exploration, Preparation, Implementation, and Sustainment Framework [34]. Proctor et al.’s reporting framework [2] could be augmented or replaced with the Workgroup for Intervention Development and Evaluation Research (WIDER) or the Template for Intervention Description and Replication (TIDieR) checklist and guide [35–37]. The behavior change technique taxonomy could be used in addition to ERIC or as a replacement, where appropriate [38]. Researchers may select frameworks based of the underlying theory, change processes, analytics level, and disciplinary credibility [29, 30]. When making decisions about combining frameworks, researchers may retain some elements we did not use here. Any use of frameworks to guide these methods should be flexible and responsive to context.

These methods have several limitations. Like other approaches to reporting and tracking research activities, these processes are time intensive and may be perceived as burdensome. This study did not allow us to estimate the time required to tracking strategies and their modifications using these methods. Future studies should consider documenting the time required to track strategies and their adaptations using these methods, to target improvements. Additional work is needed to streamline tracking to be more pragmatic [39, 40]. We refined these tracking methods based on feedback from the Implementation Support Team during weekly meetings and through a formal mid-project review and made several improvements to the methods over the course of the study. The research team refined their process for prospective tracking over the course of the study to summarize a given clinic’s incremental progress, and to guide weekly discussions of this progress. We believe this iterative improvement resulted more pragmatic tracking through an appropriate balance of prospective tracking and group discussion. These data could be collected more easily by the implementation team, and better used to guided planning efforts and implementation support. This adds to research examining the feasibility and acceptability of various approaches to tracking [10]. Our methods focused only on the delivery of implementation strategies by the study team, and did not include tracking within the clinics participating in the parent study. We did not ask study participants to complete the tracking tools presented here to minimize what was asked of the clinics, as study participation already required substantial effort on the part of participants. Additional research is needed to refine tracking tools and improve usability for practitioners and other stakeholders, including prompts for facilitated discussions and field definitions and instructions for tracking logs [10].

## Conclusions

Data collected using these methods may be used in myriad ways, such as to describe adaptations made to the originally-planned implementation strategies, or as covariates to evaluate the impact of strategies on implementation outcomes. These methods may improve assessment of implementation strategies through identifying associations between variation in strategy use and implementation outcomes and health outcomes. Data from these methods may also be used to better plan for and resource scale-up of implementation through identifying typical patterns of variation in response to context. Additional research is needed to explore methods to assess strategies and strategy modifications which most impact implementation outcomes; these methods could enhance that work [41]. Although this study did not code strategy modifications for their impact on fidelity, these methods could be expanded to track fidelity to implementation strategies by identifying core elements, developing thresholds for fidelity prior to the study, and integrating recommendations for reporting on fidelity [41, 42]. Our goal was to track the types of modifications needed and use the data to later evaluation the impact of those modifications. Future research may use these methods along with guidelines for fidelity-consistent and fidelity-inconsistent modification where core elements of the strategies are known prior to tracking efforts. Defining these components is critical for tracking strategies such as implementation facilitation and developing an implementation blueprint which are often multi-stage and widely variable in their application. Future research may further explore how to document modifications and fidelity in studies on implementation strategies’ impact.

These methods are among the first options put forth for tracking how implementation strategies are modified in implementation studies; doing so is critical for replication and scale-up of effective strategies. We present these methods to guide others seeking to document implementation strategies and modifications to these studies over the course of a research study. Future research is needed to validate and improve these methods.

## Data Availability

The data used during the current study are available from the corresponding author on reasonable request.

## Appendix

**Table 4:** Originally planned implementation support

CAP step	Specifics of implementation support	ERIC category
*Step 1.* Create an ‘SDH Team.’	Materials for clinic leaders: benefits of SDH documentation / action; leaders’ role in supporting SDH process adoption	Recruit, designate, train for leadership; orientation materials
	Materials for clinician champion: orientation, step summary materials	
Obtain leadership support.	Materials for project champion: orientation, step summary materials	
	Draft email from leadership to clinic staff alerting staff to SDH Plan	Technical assistance
Identify, orient clinic champion / study contact.	Office hours covering: (1) Orienting champions; (2) Goal setting	Identify / prepare champions; recruit, designate, train for leadership; orientation materials; peer-to-peer learning
	Check-in: Orientation	Technical assistance
*Step 2.* Identify goals.	Materials - Decision tools: Why do you want to collect SDH data? What do you hope to accomplish? What do you plan to do with the SDH data? Which patients do you want to screen? How often? For which SDH?	Goal identification / implementation blueprint
	Materials - Written recommendations / key considerations for selecting clinic goals	Goal identification; technical assistance
Identify clinic’s goals for SDH screening.	Summary of the clinic’s stated goals	Goal identification
	Office hours covering: (1) Goal setting; (2) Learning the EHR tools	Goal identification / implementation blueprint; peer-to-peer learning
Identify which patients will be screened for which SDH measures.	Check-in: Identify goals	Goal identification / implementation blueprint
*Step 3.* Create an ‘SDH Plan.’	Materials - Planning tools: SDH documentation workflow**;** SDH data review / action workflow; Workflow implementation rollout	Technical assistance
	Materials - Resource list (PRAPARE, HealthLeads, etc.)	
Create a workflow plan for SDH documentation, and (if desired) SDH data review and action.	Materials - Guides to using EHR’s SDH Data Tools: In workflows; in SDH documentation, on site or via patient portal; to review SDH; for SDH referral-making (with guidance on creating a social service resource list)	
	Materials - Pros and cons of different SDH documentation workflow options; key considerations based on other CHCs’ experience	
Create a rollout plan.	Materials - Summary of clinic’s stated workflow plan	Goal identification / implementation blueprint; technical assistance
	Check-in: Workflow development, use of workflow planning tools, rollout plan	Technical assistance
	Office hours covering: (1) Workflow planning; (2) EHR tools within workflows	Peer-to-peer learning; technical assistance
Step 4. Train clinic staff.	Materials - Orientation webinar for clinic staff; review clinic’s goals and workflow plan; include staff discussion of potential barriers / how to address them.	Educational meeting / materials; goal identification
Orient staff.		
	Materials - How to orient clinic staff to SDH documentation and action, based on other CHCs’ experiences	Educational meeting
If SDH plan changes, orient staff.	Materials - Template slides / handouts for updating staff and / or training new staff	Educational meeting; technical support
	Check-in: How to train staff	
Train new staff as needed.	Office hours covering: (1) How to train staff; (2) How to create target population reports and adoption reports	Peer-to-peer learning; technical assistance
*Step 5.* Roll out the ‘SDH Plan’	Materials - Guides: Using SDH Data Tools to review SDH documentation / action data; Using SDH documentation data to track progress; Testing workflows; PDSA cycles	Audit and feedback
Review adoption rates on a regular basis.	Check-in: Develop strategy for testing workflows, addressing barriers, rollout, review of adoption progress; how to track SDH adoption progress using data tools; how to revise workflows, rollout plan as needed	Audit and feedback; technical assistance; practice facilitation / small tests of change; tailor strategies
	Data - monthly adoption reports	Audit and feedback; tools for quality monitoring
Iterate / revise rollout, workflows as needed.	Office hours covering: How to iterate and refine workflows; other topics identified as needed by the clinics or the IST	Peer-to-peer learning; technical assistance; ongoing consultation

*Abbreviations*: *SDH* social determinants of health, *IST* Implementation Support Team, *Q & A* Questions and Answers
